# Toward a systematic grading for the selection of patients to undergo awake surgery: identifying suitable predictor variables

**DOI:** 10.3389/fnhum.2024.1365215

**Published:** 2024-05-02

**Authors:** Leonie Kram, Beate Neu, Axel Schroeder, Benedikt Wiestler, Bernhard Meyer, Sandro M. Krieg, Sebastian Ille

**Affiliations:** ^1^Department of Neurosurgery, Klinikum rechts der Isar, Technical University of Munich, School of Medicine, Munich, Germany; ^2^Department of Neurosurgery, Heidelberg University Hospital, Ruprecht-Karls-University of Heidelberg, Heidelberg, Germany; ^3^Section of Diagnostic and Interventional Neuroradiology, Department of Radiology, Klinikum rechts der Isar, Technical University of Munich, School of Medicine, Munich, Germany; ^4^TUM-Neuroimaging Center, Klinikum rechts der Isar, Technical University of Munich, School of Medicine, Munich, Germany

**Keywords:** awake craniotomy, language eloquence, glioma, preoperative language status, multinomial logistic regression

## Abstract

**Background:**

Awake craniotomy is the standard of care for treating language eloquent gliomas. However, depending on preoperative functionality, it is not feasible in each patient and selection criteria are highly heterogeneous. Thus, this study aimed to identify broadly applicable predictor variables allowing for a more systematic and objective patient selection.

**Methods:**

We performed post-hoc analyses of preoperative language status, patient and tumor characteristics including language eloquence of 96 glioma patients treated in a single neurosurgical center between 05/2018 and 01/2021. Multinomial logistic regression and stepwise variable selection were applied to identify significant predictors of awake surgery feasibility.

**Results:**

Stepwise backward selection confirmed that a higher number of paraphasias, lower age, and high language eloquence level were suitable indicators for an awake surgery in our cohort. Subsequent descriptive and ROC-analyses indicated a cut-off at ≤54 years and a language eloquence level of at least 6 for awake surgeries, which require further validation. A high language eloquence, lower age, preexisting semantic and phonological aphasic symptoms have shown to be suitable predictors.

**Conclusion:**

The combination of these factors may act as a basis for a systematic and standardized grading of patients’ suitability for an awake craniotomy which is easily integrable into the preoperative workflow across neurosurgical centers.

## 1 Introduction

Since the early 20th century, direct electrical stimulation (DES) during awake surgery is used to localize and study language and speech in patients (Rahimpour et al., 2019). Whilst this method was initially applied for mapping epileptic foci, it soon was extended to resections of brain tumors in eloquent areas (Penfield and Roberts, 1959; Ojemann et al., 1989; Surbeck et al., 2015).

With technological and methodological advances, awake language mapping became the gold standard for balancing the preservation of language function and the extent of resection in patients with brain tumors (Mandonnet et al., 2010). Moreover, growing evidence supported a variable and individual distribution of language functions across a wide-spread cortical and subcortical network (Duffau et al., 2014; Chang et al., 2015). This inter-individual variability in combination with potential tumor-induced functional reorganization (Thiel et al., 2005; Krieg et al., 2013; Wang et al., 2013; Ille et al., 2019) underlines the necessity of thoroughly testing language localization in patients with brain tumors in critical areas.

Still, awake language mapping is not feasible in every patient. It requires patients to perform language tasks while stimulation elicits transient disruptions of the language network resulting in audible language errors (Talacchi et al., 2013b). Thus, patients need to have sufficient language skills to complete these tasks. Whilst no standardized guidelines are available, some studies suggest a cut-off at 25% errors during baseline or 50% errors in all modules of a standardized test battery as a contraindication for awake mapping (Picht et al., 2006; Hervey-Jumper and Berger, 2016). Still, it is often not mentioned how these thresholds were derived nor were different error types differentiated. Moreover, no consensus exists about what constitutes sufficient language skills and which role different aphasia types and symptoms play. Different language tasks and testing batteries may be employed during DES awake language mapping (De Witte et al., 2015; Martin-Monzon et al., 2022). As these can test heterogeneous language abilities, each test requires sufficient task-specific skills. Since object naming is one of the most common intraoperative language tests (Martin-Monzon et al., 2022), we focused our analysis on preoperative object naming performance as an indicator for patient’s suitability to undergo awake surgery. During naming tasks, impairments may comprise phonological and semantic paraphasias, neologisms, no responses, or circumlocutions (Fridriksson et al., 2009; Meier et al., 2020) in various combinations and degrees of severity, impacting the patient’s suitability for awake surgeries with regard to the intraoperative analysis.

In addition, the location of a tumor in language eloquent areas is a key component in the decision process for or against an awake surgery. Still, the structural language network comprises a multitude of subcortical tracts and cortical areas (Chang et al., 2015; Friederici, 2017) covering a large proportion of the left hemisphere which complicates the differentiation of the level of language eloquence. A recently published three-tier grading system allows to define the grade of language eloquence in a standardized and systematic way (Ille et al., 2021). Based on cortical and subcortical tumor localization as well as clinical presence of preexisting aphasia, the level of language eloquence is defined, which has shown to be higher in awake surgery cases (Ille et al., 2021).

Moreover, awake surgeries require a large multidisciplinary team, consequently increasing timely and staff effort, as well as may cause additional psychological strain for patients and pose the risk of seizures (Talacchi et al., 2013a). This further highlights the necessity of careful and systematic patient selection criteria.

Typically, whilst many – especially large – neurosurgical centers perform preoperative functional imaging, this may not be available in every clinic. Since the aim of the present exploratory study was to develop a general, standardized support for the indication of awake or asleep procedures, no preoperative non-invasive functional imaging or stimulation data was used.

We ascertained whether the severity of different aphasic features during naming and patient-specific characteristics in combination with a recently published standardized grading of language eloquence (Ille et al., 2021) are suitable variables for patient selection.

## 2 Materials and methods

### 2.1 Ethics

This study was approved by the local ethics committee of the Institutional Review Board of Technical University of Munich (reference number: 192/18). Moreover, it followed the guidelines of the Declaration of Helsinki.

### 2.2 Patient selection

**Figure 1:**
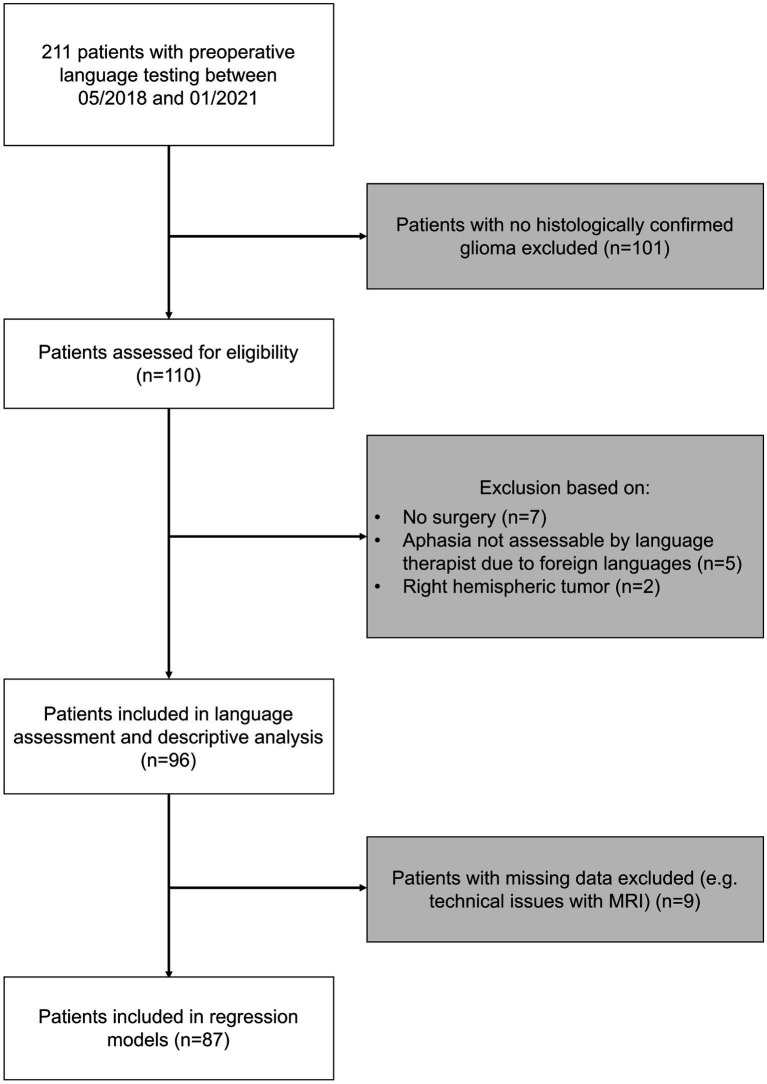
Selection process. The flowchart describes the selection process of patients included in this study. Exclusion criteria highlighted in gray. Number (*n*) of patients excluded as well as considered are provided for each selection step.

We performed a post-hoc analysis of prospectively enrolled patients with left-hemispheric and suspected language eloquent brain tumors who underwent preoperative video-recorded standardized object naming in our department between May 2018 and January 2021. This cohort partly overlaps with the cohort of Ille et al. (2021) in which the language eloquence grading was first established. All patients needed to be at least 18 years old. Patients with cochlear implants or pacemakers were excluded. Only patients with histologically confirmed glioma were considered for analysis. Moreover, patients who did not undergo surgery were excluded. In addition, patients needed to be proficient speakers of German to allow for an in-depth analysis of their language status. Handedness was checked with the Edinburgh Handedness Inventory (Oldfield, 1971). An overview of the selection process is provided in Figure 1.

### 2.3 Language assessment

Language performance was assessed by a trained speech and language therapist (SLT, L.K.) based on video recordings of a standardized routine object naming task (Krieg et al., 2017; Ille et al., 2021). During this analysis, the SLT was blinded to type of surgery, tumor entity and localization. The task comprised black-and-white drawings of 80 common objects. Naming can provide valuable insight into the patients’ productive language abilities as naming tasks are commonly included in standardized language assessment tools (Huber et al., 1983; Kertesz, 2007). This post-hoc in-depth analysis of the video recordings allowed to identify the following types of language errors: automated language elements (e.g., perseveration, phrase), paraphasia (e.g., semantic or phonological paraphasia, conduit d’approche, conduit d’écart), neologisms (semantic or phonological neologisms), and word finding difficulties. Moreover, the overall expressive aphasia severity was rated by the SLT (0 = no aphasia to 5 = extremely severe aphasia).

### 2.4 Magnetic resonance imaging

A standardized MRI protocol (Sollmann et al., 2016, 2018) was followed which is routinely performed prior to surgery with a 3 T MRI scanner (Achieva dStream or Ingenia; Philips Healthcare, Best, Netherlands) with an 8-or 32-channel phased-array head coil in the department of neuroradiology. Structural MRI scans with at least a three-dimensional T1-weigthed gradient echo sequence (with and without contrast agent) and a diffusion tensor imaging sequence with 32 diffusion directions were derived for each patient.

### 2.5 Language eloquence classification

**Figure 2:**
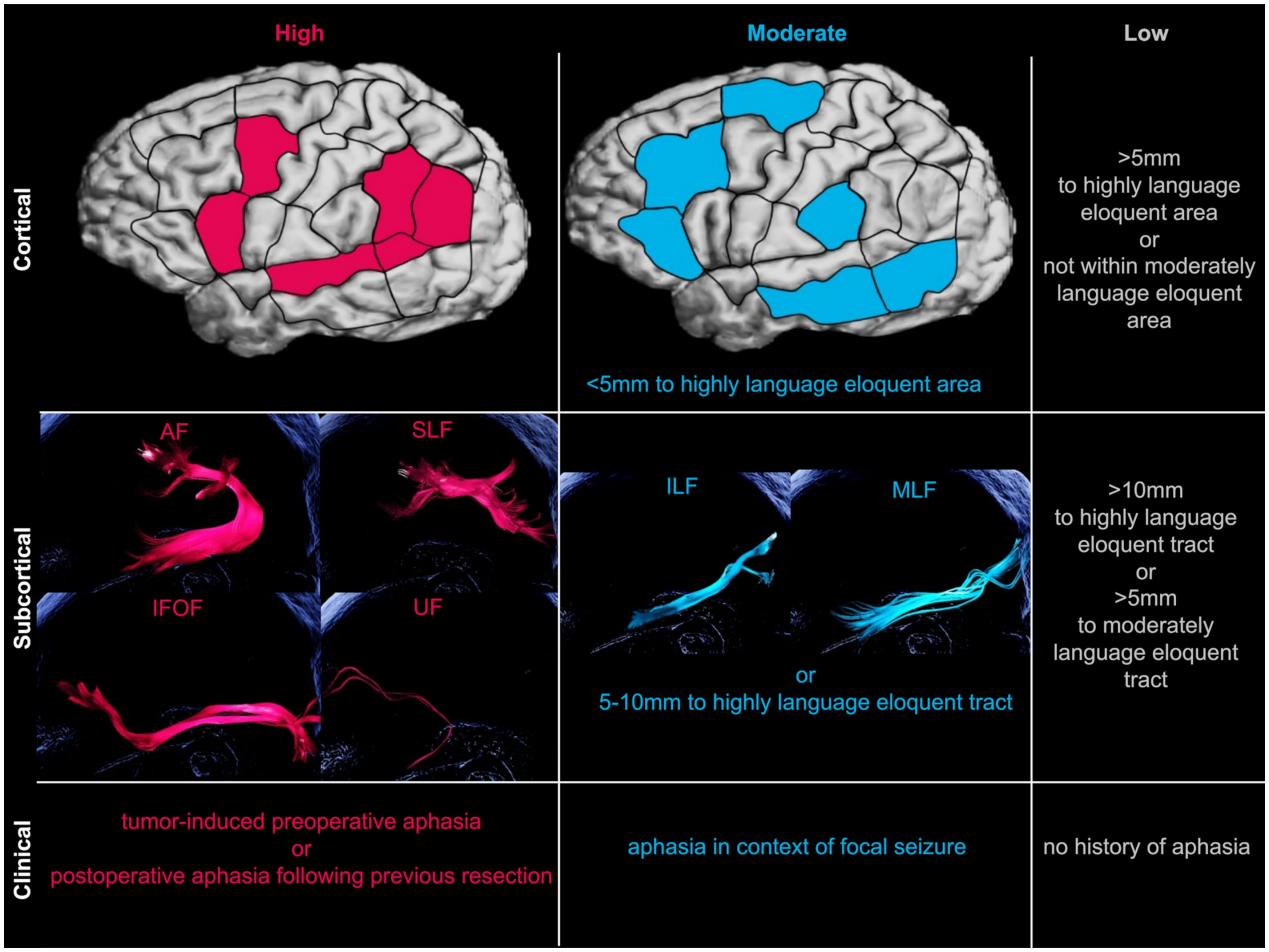
Schematic overview of language eloquence classification. Classifying language eloquence levels into high (pink), moderate (blue), or low (gray) levels based on cortical, subcortical, and clinical characteristics (Ille et al., 2021). Exemplary illustration of respective subcortical language eloquent fibertracts created with deterministic tractography software package for neurosurgical applications (Brainlab AG, Munich, Germany). *AF, arcuate fasciculus; IFOF, inferior fronto-occipital fasciculus; ILF, inferior longitudinal fasciculus; MLF, middle longitudinal fasciculus; SLF, superior longitudinal fasciculus; UF, uncinate fasciculus.

In order to directly compare language eloquence across patients, a recently published standardized three-tier system was applied (Ille et al., 2021). This classification, developed for scientific analysis of tumor localizations, allows to classify the overall low (0–2), moderate (3–5) and high (6–9) level of language eloquence based on cortical, subcortical and clinical characteristics (Figure 2). Tumor localization within highly or moderately language eloquent cortical areas and subcortical white matter pathways or within a predefined distance to these, respectively, is attributed with 0 to 3 points. This is purely based on the preoperative MRI scans, with no additional processing or analysis steps. Additionally, if patients present with tumor-induced preoperative aphasia or language impairments following a previous resection, two points for high clinical language eloquence are added. If, however, aphasia manifested in context of focal seizures, one point is given for moderate clinical language eloquence. The sum of all these components finally determines the overall language eloquence level ranging from 0 to 9.

### 2.6 Statistical analysis

Statistical analyses were performed with R (R Core Team, 2022; Rstudio Team, 2022), plots created with ggplot2 R package (Wickham, 2016). A *p-*value <0.05 was considered statistically significant. Binomial logistic regression was carried out to ascertain the effects of age, sex, the number of manifestations of the language impairment categories (automated language elements, paraphasias, word finding difficulties, neologisms), language eloquence category (low, moderate, high) and histologically confirmed WHO CNS grade (1–4) on the likelihood of being operated awake compared to being operated asleep. Multicollinearity and linear relationship between logit transformation of dependent variable and continuous independent variables were checked in advance.

Stepwise backward variable selection based on the Akaike Information Criterion (AIC) (Akaike, 1973/1998) using the *stepAIC* function of the MASS package in R (Venables and Ripley, 2002) was applied to identify predictor variables. This method maintains a good model performance while reducing the number of predictor variables (Sanchez-Pinto et al., 2018).

Subsequently, a thorough descriptive and graphical analysis of significant predictor variables was performed. Moreover, to evaluate discriminative abilities of the continuous predictor variables, the respective area under the curve (AUC) and receiver operating characteristic (ROC) curves were compared (Robin et al., 2011). If applicable, Youden’s J statistic was employed to determine the optimal cut-off value (Youden, 1950).

## 3 Results

### 3.1 Patient and tumor characteristics

This study included 96 glioma patients with a mean age of 57.8 ± 14.3 (range: 22–85) years who performed a preoperative video-recorded standardized object naming between May 2018 and January 2021. Of these, 54 were male (56.2%). Histopathology confirmed a glioma in all cases, 28 of which were tumor recurrences or progresses (29.2%). The largest proportion of patients presented with a WHO CNS grade 4 tumor (65.6%). 22.9% of patients had a confirmed WHO CNS grade 3, 9.4% a CNS grade 2 and 2.1% of patients a CNS grade 1 tumor. Tumor locations predominantly comprised the left hemisphere (93 cases, 96.9%). Three cases presented with a bilateral glioma. In these cases, however, the left hemispheric part was resected and consequently, only the left hemispheric tumor location considered for language eloquence definition. Four patients were left-handed (4.4%), seven ambidextrous (7.7%) and 80 right-handed (87.9%). Overall, 25 patients received an awake surgery (26.0%) whereas 71 patients underwent asleep tumor resection (74.0%). Across all awake cases 80.0% and across all asleep cases 91.5% were high-grade gliomas (WHO CNS grade 3 and 4).

### 3.2 Language status and eloquence

Overall, 23 patients did not present with an aphasia prior to surgery, whilst 73 patients showed at least minimal aphasic symptoms based on the 80-item object naming task. Moreover, across surgery types, 12 cases had a low (13.8%), 41 a moderate (47.1%) and 34 a high language eloquence level (39.1%). Table 1 summarizes the absolute and relative frequencies for each aphasia severity level (0–5) and language eloquence level (0–9) as well as descriptive for each language error type across all patients (total) and for each surgery type.

**Table 1:** Overview of absolute and column-wise relative frequencies of aphasia severity and language eloquence levels per surgery type as well as the descriptives [mean ± standard deviation (range)] for each language error type.

		Surgery type		
		Asleep (*n* = 71)	Awake (*n* = 25)	Total (*n* = 96)
Aphasia severity	0	15 (21.1%)	8 (32.0%)	23 (24.0%)
	1	15 (21.1%)	6 (24.0%)	21 (21.9%)
	2	20 (28.2%)	4 (16.0%)	24 (25.0%)
	3	13 (18.3%)	3 (12.0%)	16 (16.7%)
	4	1 (1.4%)	4 (16.0%)	5 (5.2%)
	5	7 (9.9%)	0 (0.0%)	7 (7.3%)
Language eloquence	Missing	8	1	9
	0	6 (9.5%)	2 (8.3%)	8 (9.2%)
	1	0 (0.0%)	0 (0.0%)	0 (0.0%)
	2	4 (6.3%)	0 (0.0%)	4 (4.6%)
	3	15 (23.8%)	2 (8.3%)	17 (19.5%)
	4	7 (11.1%)	0 (0.0%)	7 (8.0%)
	5	13 (20.6%)	4 (16.7%)	17 (19.5%)
	6	7 (11.1%)	5 (20.8%)	12 (13.8%)
	7	2 (3.2%)	1 (4.2%)	3 (3.4%)
	8	6 (9.5%)	9 (37.5%)	15 (17.2%)
	9	3 (4.8%)	1 (4.2%)	4 (4.6%)
Language error type^*^	AL	2.3 ± 4.1 (0–28)	1.5 ± 2.5 (0–8)	2.1 ± 3.7 (0–28)
	P	4.2 ± 3.6 (0–19)	6.2 ± 7.1 (0–27)	4.7 ± 4.8 (0–27)
	N	0.9 ± 2.6 (0–14)	1.1 ± 3.8 (0–18)	1.0 ± 2.9 (0–18)
	S	0.1 ± 0.3 (0–2)	0.0 ± 0.2 (0–1)	0.1 ± 0.3 (0–2)
	WF	15.2 ± 21.3 (0–80)	9.8 ± 14.1 (0–55)	13.8 ± 19.8 (0–80)

*AL, automated language elements; P, paraphasias; N, neologisms; S, suprasegmental errors; WF, word finding difficulties.

Since frequently a cut-off value at a minimum of 25% of errors during baseline naming testing for non-eligibility of an awake language mapping is proposed (Hervey-Jumper and Berger, 2016), the language status and surgery type allocation were descriptively compared to this established cut-off value. Across surgery types 63 patients would be considered eligible and 33 non-eligible according to the 25% rule of thumb. Of these 63 eligible patients, 98.4% had no, minimal or light aphasic symptoms while of the 33 non-eligible patients, 81.8% showed moderate, severe or extremely severe expressive aphasic symptoms during the naming task. According to this rule, 63.4% of asleep and 72.0% of awake surgery cases would have been considered eligible for an awake craniotomy. Moreover, 57.1% of the awake surgery cases who would have been considered non-eligible according to the frequently used cut-off value, had a language eloquence level of at least 6. Thus, in these cases language eloquent tumor location may have supported the decision for an awake surgery.

Across all 25 awake surgeries, no difficulties due to poor intraoperative performance were reported, in 96% of these surgeries awake language testing was feasible. A single non-aphasic case was reported whose anesthesia did not wear off properly which limited the patient’s intraoperative performance capabilities already in the beginning of the awake language testing phase. Moreover, two cases showed increased pain levels during the course of the surgery. The pain medication impacted language production abilities in one of these cases while the awake testing needed to be stopped due to strong pain levels in the other case.

### 3.3 Binomial logistic regression

Multicollinearity analysis indicated a high correlation between the number of paraphasias and the number of neologisms. Since paraphasias are the more prevalent aphasic symptom and the aim of this study was to identify clinically relevant predictor variables, neologisms were not included in subsequent analysis.

Stepwise backward variable selection based on AIC indicated only three important predictor variables in the given model: number of paraphasias, language eloquence category, and age. The following variables did not add significant information to the model: sex, WHO CNS grade, automated language elements, and word finding difficulties. Since, moreover, only the contrast of high compared to moderate language eloquence was statistically significant, but not the contrast between high and low, the latter was dropped from the final model.

Thus, the final logistic regression model evaluated the effects of age, number of paraphasias and moderate compared to high language eloquence on the likelihood that a patient receives awake or asleep surgery.

The final logistic regression model was statistically significant [*Χ*^2^(3) = 26.1, *p* < 0.001]. The model explained 41.9% (Nagelkerke *R*^2^) of the variance in the type of surgery. Patients with a high language eloquence were 6.5 times more likely to be operated awake than patients with a moderate language eloquence (OR = 6.5, 95%CI[1.9, 26.2]). Increasing age was associated with a decreased likelihood (OR = 0.9, 95%CI[0.9, 1.0]), whilst a higher number of paraphasias was associated with an increased likelihood of receiving an awake surgery (OR = 1.2, 95%CI[1.0, 1.3]).

### 3.4 Analysis of important predictor variables

**Figure 3:**
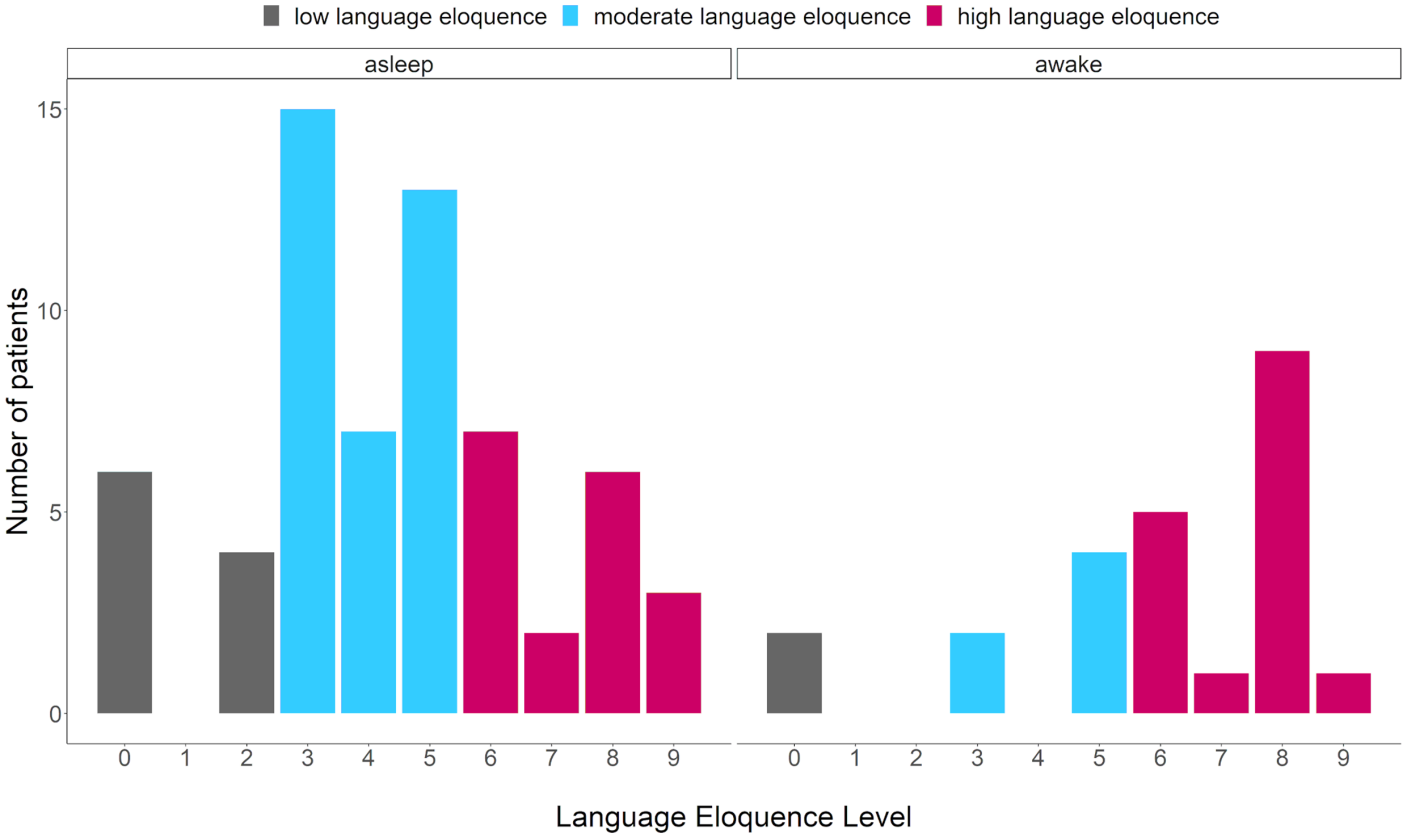
Comparison of number of patients presenting with a specific language eloquence level. Eloquence ranging from 0 to 9, colors indicating the language eloquence category (gray = low, blue = moderate, pink = high) per surgery type (asleep, awake).

Firstly, the prevalence of language eloquence category per surgery type was compared (Figure 3). The absolute and relative frequencies of language eloquence level for each surgery type as well as across patients are summarized in Table 2. As the regression analysis indicated, a high language eloquence was associated with a higher likelihood of being operated awake. Consequently, our results suggest a language eloquence level of at least 6 for awake surgery indication.

**Table 2:** Overview of absolute and relative frequencies of language eloquence categories per surgery type.

Language eloquence category	Surgery type		
	Asleep (*n* = 63)	Awake (*n* = 24)	Total (*n* = 87)
Low	10 (15.9%)	2 (8.3%)	12 (13.8%)
Moderate	35 (55.6%)	6 (25.0%)	41 (47.1%)
High	18 (28.6%)	16 (66.7%)	34 (39.1%)

**Figure 4:**
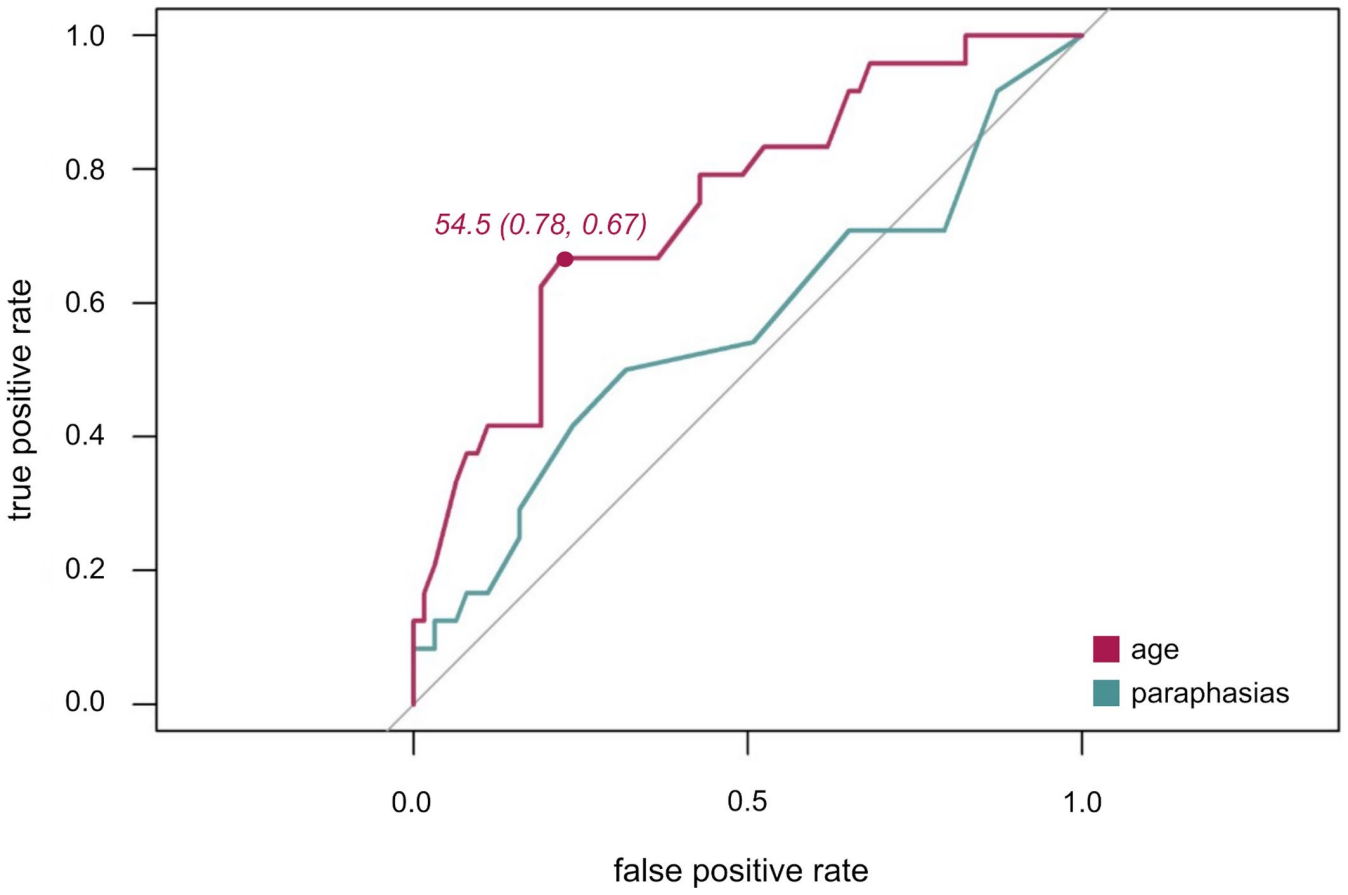
ROC curves for age (purple) and paraphasias (green). ROC plots illustrating the true positive rate (sensitivity) plotted against the false positive rate (1-specificity) with Youden index for age (sensitivity, specificity).

Secondly, the discrimination ability of age for awake compared to asleep surgery was assessed. In the ROC curve (Figure 4), the true positive (sensitivity) in relation to the false positive rate (1-specificity) are plotted for the ability of age to predict type of surgery with an AUC of 0.7. Youden’s J statistic identified an optimal cut-off value of 54.5 years. Still, nine patients of the awake surgery group were at least 58, the oldest patient of the awake group was 75 years old.

Thirdly, the discrimination ability of number of paraphasias for awake compared to asleep surgery was ascertained. As indicated by the ROC (Figure 4) and an AUC of 0.6, this variable alone is not a suitable predictor variable for differentiating between awake and asleep surgeries. Thus, no optimal cut-off was defined for this variable.

Finally, language eloquence categories per surgery type were evaluated for two separate patient groups defined by the optimal age cut-off value: higher age group (≥55 years) and lower age group (≤ 54 years). The results are summarized in Table 3. Overall, 77.8% of asleep surgery cases were in the higher age group while 66.7% of awake surgery cases were in the lower age group. Across surgery types, 65.5% of patients were in the higher age group.

**Table 3:** Overview of absolute and column-wise relative frequencies of language eloquence categories (low, moderate, high) per surgery type (asleep, awake, total) for each age group (high, low) separately.

Age group	Language eloquence category	Surgery type		
		Asleep (*n* = 63)	Awake (*n* = 24)	Total (*n* = 87)
High (≥ 55 years)	Low	9 (18.4%)	0 (0.0%)	9 (15.8%)
	Moderate	26 (53.1%)	3 (37.5%)	29 (50.9%)
	High	14 (28.6%)	5 (62.5%)	19 (33.3%)
Low (≤ 54 years)	Low	1 (7.1%)	2 (12.5%)	3 (10.0%)
	Moderate	9 (64.3%)	3 (18.8%)	12 (40.0%)
	High	4 (28.6%)	11 (68.8%)	15 (50.0%)

## 4 Discussion

Awake surgeries remain the standard of care to enhance quality of life and general prognosis in the neurooncological treatment of patients with low-and high-grade language eloquent gliomas (Mandonnet et al., 2010; De Witt Hamer et al., 2012). Whilst this assumption is widely accepted, no consensus and standardized recommendations exist for determining which patient is suitable for an awake craniotomy. Rather, this decision is highly subjective and can vary considerably depending on the neurosurgical center. For this reason, this study aimed to identify objective suitable factors for patient selection. Stepwise backward selection based on Akaike Information Criterion confirmed that a higher number of paraphasias, a lower age, and a high level of language eloquence were suitable indicators for an awake surgery in our glioma cohort. Subsequent descriptive and ROC-analyses indicated a cut-off value of ≤54 years and a language eloquence level of at least 6 for awake surgeries, which require further validation. The present results may add valuable insights into which factors should be considered and may act as a basis for subsequent large-scale, multicentric trials.

### 4.1 Preoperative aphasia and importance of particular naming error types

DES-based language mapping establishes a causal link between the directly stimulated cortical site and language function. In order to localize cortical language sites, patients need to have sufficient language skills to perform these tasks. The present results indicate that even patients with moderate or severe language deficits, who would be excluded according to established cut-off criteria such as the maximum of 25% of errors to be considered eligible for an awake procedure (Hervey-Jumper and Berger, 2016), could undergo DES-based language mappings even if the number of items included for baseline naming was limited. All awake language testing was reported to be feasible, no early termination due to a patient’s language capabilities was necessary.

The results of the multinomial logistic regression analysis indicate that the number of paraphasias was the only significant predictor variable of the analyzed language error types. Patients with a higher number of paraphasias were more likely to be operated awake than asleep. These word substitutions semantically related to the target item or phonological errors frequently manifest in aphasic patients during naming tasks as implemented in this study (Meier et al., 2020). The presence of aphasic symptoms reflects the clinical language eloquence a patient has. The higher pronounced the aphasic deficit the more likely a tumor is language eloquent which would provide an indication for an awake surgery. This additionally reflects the importance of including a clinical language component into a language eloquence grading as proposed by Ille et al. (2021).

Still, within the present cohort, no clear cut-off value could be defined and the overall predictability of paraphasias alone was low as indicated by the ROC curve (Figure 4). Thus, a specific range rather than one cut-off value may be indicative of awake surgeries. Whilst a certain number of aphasic symptoms increases the suitability of a patient as clinical symptoms indicate a clinical language eloquence, a too high number preventing an adequate intraoperative performance on language tasks would decrease the suitability of patient. This would extend previous suggestions of one definite cut-off value for contraindicating DES-based language mapping (Picht et al., 2006; Hervey-Jumper and Berger, 2016). At the same time, this would explain why paraphasias, as opposed to word finding difficulties, were a predictive error type. They former occurred with a range of 0 to 27 errors out of 80 named objects within the awake group while word finding difficulties manifested a lot more frequently with a range of 0–55 in the awake and 0–80 in the asleep group. Accordingly, language skills and preexisting aphasia provide valuable information about the suitability of a patient to be operated awake. Thus, defining adequate standardized cut-off minimum and maximum values in subsequent prospective studies which assess pre-and intraoperative language performance systematically would be highly valuable.

### 4.2 Differentiation ability of standardized language eloquence classification

The complexity of the cortical and subcortical language network and the heterogeneity of methodologies and techniques used to identify eloquence, complicate the exact definition and comparison of language eloquence across patients and studies. To allow for a direct and systematic comparison of language eloquence, we utilized a standardized language eloquence grading assigning a high, moderate and low language eloquence level to each patient (Ille et al., 2021). As expected, our results confirmed that a high language eloquence increased the likelihood of an awake surgery compared to moderate eloquence. Whilst the largest proportion of awake surgery cases had a high language eloquence (66.7%), only 28.6% of the asleep surgery cases presented with a high eloquence. This is in line with findings of Ille et al. (2021) who reported that most patients who had an awake craniotomy also had a high language eloquence. Moreover, around half of the asleep (55.6%) and a quarter of awake surgery cases (25.0%) had a moderate language eloquence level. Simultaneously, only two patients of the awake and ten of the asleep surgery group presented with a low language eloquence. These limited patient numbers with low eloquence may explain why differentiating between low and high eloquence could not predict the type of surgery.

Overall, this systematic and standardized classification enabled to directly compare language eloquence based on the integration of structural imaging and functional preoperative status. Preoperative functional mapping is frequently performed prior to an awake surgery. Still, neuroimaging or stimulation paradigms differ considerably (Agarwal et al., 2019; Haddad et al., 2021; Ille and Krieg, 2021) and yet no consensus about the suitability of one method over the other exists. This restricts the broad applicability of one of these methods across multiple centers. Moreover, some even suggest not to base the decision for or against an awake surgery on functional imaging (Hervey-Jumper et al., 2015; Gogos et al., 2020). Thus, this standardized classification may offer an easily employable alternative for preoperative assessment of language eloquence. Overall, this eloquence grading may provide valuable information about the indication for an awake surgery as supported by the present study’s results.

### 4.3 Implications of age for awake surgeries

Multiple studies report the necessity to advance and evaluate treatment approaches for the elderly patient cohort (Braun and Ahluwalia, 2017; Yuen et al., 2022). It remains unclear whether awake surgery is feasible in higher aged patients. For instance, a recent meta-analysis comprising 134 patients reported a mean pooled age of only 46.9 years (Zhang et al., 2020). Some even suggest that an age of above 65 years would be a strict contraindication for an awake craniotomy (Bertani et al., 2009). At the same time, Hervey-Jumper et al. (2015) conducted awake surgeries in patients up to an age of 84 years.

Since particularly high-grade tumors are more prevalent in elderly patients (Ostrom et al., 2022) and these more aggressive tumor entities are known to elicit worse functional deficits, the entity rather than the age may frequently impact the decision for or against an awake procedure. Still, across both surgery types, the largest proportion of patients presented with high-grade gliomas. As the multicollinearity analysis did not indicate a strong correlation between tumor entity and age, and, moreover, tumor entity was not a predictor for the type of surgery, age rather than the entity itself seemed to be indicative of the type of surgery.

Whilst the present study did not aim for setting a specific cut-off value widely applicable, our results suggest a cut-off value of 54 years or younger as suitable for awake surgeries in our patient cohort. Across surgery types a higher proportion of patients were aged 55 or above. At the same time, a higher number of awake surgery patients was in the lower age group whilst most of the asleep surgery cases were in the higher age group. Nevertheless, nine of the awake surgery cases were between 58 and 75 years. Across these nine patients, awake testing was feasible, no early termination of the awake testing phase reported. However, across all cases the younger patients were, the more likely they had an awake craniotomy. Still, for the present analysis, we did not evaluate the postoperative outcome nor how well patients could perform during the awake phase. Grossman et al. (2013) showed that even in elderly patients (>65 years) awake mapping was feasible and lead to similar outcomes as in a younger cohort. Consequently, it would be highly valuable to consider the impact of age on these factors and assess how well older patients can tolerate awake surgeries in subsequent prospective, large-scale studies. Simultaneously, these studies could systematically ascertain whether any other age-related comorbidities may impact the decision of awake surgery indication and the feasibility or performance capabilities of patients, and consequently, need to be considered within a widely applicable grading tool.

### 4.4 Limitations and perspectives

Since no video-recordings of the intraoperative awake procedure were available for this post-hoc analysis, the present results do not consider how well patients performed naming tasks nor the naming accuracy intraoperatively. Systematically evaluating the intraoperative language performance may allow for a more detailed assessment of the predictive ability of preoperative language skills, error types, and aphasia severity. Still, awake testing was feasible in 96% of the 25 cases selected, only a single patient could not be tested adequately during DES-based language mapping as his anesthesia did not wear off properly. Moreover, no complications apart from two cases with increased pain levels or decreased language performance due to pain medication during the course of the language testing were reported. Thus, the patients’ language capabilities seemed to be adequate for the planned procedure. At the same time, prospective studies are warranted to thoroughly and systematically document and evaluate the patient’s intraoperative performance and naming accuracy. By systematically comparing pre-and intraoperative performance, the impact of preexisting aphasic deficits on naming accuracy and overall performance during awake surgery can be assessed thoroughly.

Additionally, due to the post-hoc nature of the present study, the evaluation of preoperative language abilities could only be based on the standardized object naming task which only allows the stratification on the basis of the patients’ object naming abilities. Language is a highly complex and dynamic function (Duffau, 2016; Friederici, 2017). Since language deficits can affect a multitude of linguistic processes and modalities, diagnostical tools cover a multitude of different linguistic functions across linguistic modalities to assess this complexity (Huber et al., 1983; Kertesz, 2007). Moreover, next to language, ample cognitive abilities contribute to maintaining the patients’ quality of life. Therefore, more and more test paradigms are introduced which, for instance, allow mapping of visuo-spatial, emotional, or executive abilities as well as memory or calculation (Ruis, 2018; Lemaitre et al., 2022). What is even more, due to the interaction between cognitive and language networks particularly in context of neural adaption processes (Brownsett et al., 2014; Hartwigsen and Volz, 2021), preservation of cognitive networks may support the compensation of language network disruptions and associated impairments. Thus, subsequent prospective and large-scale studies may benefit from thorough preoperative neuropsychologic and language testing next to the object naming task to provide a more complete picture of the preserved and impaired language abilities. Still, this standardized naming task was selected since object naming is one of the most prevalent intraoperative task applied during DES-based surgeries (Martin-Monzon et al., 2022). Hence, to assess whether language abilities are sufficient, evaluating the patient’s preoperative performance in this task may be even more relevant than the performance in classic language or neuropsychological diagnostical tools if the results are used to inform a widely applicable systematic grading. Moreover, all analyses were based on patients treated within one neurosurgical center. Consequently, large-scale, multicenter trials should be performed to ascertain whether the identified factors are valid, objective, and reproducible predictors across neurosurgical departments. Additionally, due to the limited sample size particularly in the awake cohort, it was statistically not feasible to split off part of the data set for validation of its general applicability. For the same reason, no machine learning approaches such as classification trees were implemented. Subsequent studies with larger sample sizes may implement such classification and validation approaches.

Preoperative functional imaging and modulation methods are frequently utilized to guide surgical planning and resection and have shown to be highly valuable for preserving language function (Ille et al., 2016; Castellano et al., 2017; Haddad et al., 2021). Whilst functional data could provide critical insight into the necessity of an awake language mapping, the heterogeneity of methodologies, testing designs, and techniques employed makes it challenging to build a widely applicable grading for awake surgeries. Still, a systematic and standardized grading based on preoperative language abilities and language eloquence as well as more general factors such as age could be used in addition to functional imaging across centers, adding a more objective perspective into the decision process.

## 5 Conclusion

Selecting suitable candidates for awake craniotomies remains challenging. Thus far, no standardized, objective classification system supports this decision process. A high language eloquence, lower age, and preexisting semantic as well as phonological aphasic symptoms have shown to be suitable predictors of the standard of care in language eloquent glioma patients. Consequently, the combination of these factors may act as a basis for developing a systematic and standardized grading for patients’ suitability for an awake craniotomy which is easily integrable into the preoperative workflow across neurosurgical centers.

## Data availability statement

The data presented in this study is available in Table 1, 2 and 3. Due to privacy restrictions of our clinical data, individual MRI and video data cannot be made publicly available. All data presented in this study is available upon reasonable request.

## Ethics statement

This study was approved by the local ethics committee of the Institutional Review Board of Technical University of Munich (reference number: 192/18). Moreover, it followed the guidelines of the Declaration of Helsinki. The study was conducted in accordance with the local legislation and institutional requirements. The participants provided their written informed consent to participate in this study.

## Author contributions

LK: Conceptualization, Formal analysis, Investigation, Methodology, Visualization, Writing – original draft, Writing – review & editing. BN: Formal analysis, Methodology, Writing – review & editing. AS: Investigation, Writing – review & editing. BW: Writing – review & editing. BM: Resources, Writing – review & editing. SK: Conceptualization, Project administration, Resources, Supervision, Writing – review & editing. SI: Conceptualization, Methodology, Project administration, Supervision, Writing – review & editing.

## References

[ref1] Agarwal S. Sair H. I. Gujar S. Pillai J. J. (2019). Language mapping with fMRI: current standards and reproducibility. Top. Magn. Reson. Imaging 28, 225–233. doi: 10.1097/RMR.0000000000000216 31385902

[ref2] Akaike H. (1973/1998). “Information theory and an extension of the maximum likelihood principle” in Selected papers of Hirotugu Akaike. eds. Parzen E. Tanabe K. Kitagawa G. (New York: Springer), 199–213.

[ref3] Bertani G. Fava E. Casaceli G. Carrabba G. Casarotti A. Papagno C.. (2009). Intraoperative mapping and monitoring of brain functions for the resection of low-grade gliomas: technical considerations. Neurosurg. Focus. 27:E4. doi: 10.3171/2009.8.FOCUS09137, PMID: 19795953

[ref4] Braun K. Ahluwalia M. S. (2017). Treatment of glioblastoma in older adults. Curr. Oncol. Rep. 19:81. doi: 10.1007/s11912-017-0644-z 29075865

[ref5] Brownsett S. L. E. Warren J. E. Geranmayeh F. Woodhead Z. Leech R. Wise R. J. S. (2014). Cognitive control and its impact on recovery from aphasic stroke. Brain 137, 242–254. doi: 10.1093/brain/awt289, PMID: 24163248 PMC3891442

[ref6] Castellano A. Cirillo S. Bello L. Riva M. Falini A. (2017). Functional MRI for surgery of gliomas. Curr. Treat. Options Neurol. 19:34. doi: 10.1007/s11940-017-0469-y 28831723

[ref7] Chang E. F. Raygor K. P. Berger M. S. (2015). Contemporary model of language organization: an overview for neurosurgeons. J. Neurosurg. 122, 250–261. doi: 10.3171/2014.10.JNS132647, PMID: 25423277

[ref8] De Witt Hamer P. C. Robles S. G. Zwinderman A. H. Duffau H. Berger M. S. (2012). Impact of intraoperative stimulation brain mapping on glioma surgery outcome: a meta-analysis. J. Clin. Oncol. 30, 2559–2565. doi: 10.1200/JCO.2011.38.4818, PMID: 22529254

[ref9] De Witte E. Satoer D. Robert E. Colle H. Verheyen S. Visch-Brink E.. (2015). The Dutch linguistic intraoperative protocol: a valid linguistic approach to awake brain surgery. Brain Lang. 140, 35–48. doi: 10.1016/j.bandl.2014.10.011, PMID: 25526520

[ref10] Duffau H. (2016). “White matter pathways in the human” in Neurobiology of language. eds. Hickok G. Small S. L. (London, UK; San Diego, USA; Waltham, MA, USA; Oxford, UK. Academic Press, Elsevier), 129–137.

[ref11] Duffau H. Moritz-Gasser S. Mandonnet E. (2014). A re-examination of neural basis of language processing: proposal of a dynamic hodotopical model from data provided by brain stimulation mapping during picture naming. Brain Lang. 131, 1–10. doi: 10.1016/j.bandl.2013.05.011, PMID: 23866901

[ref12] Fridriksson J. Baker J. M. Moser D. (2009). Cortical mapping of naming errors in aphasia. Hum. Brain Mapp. 30, 2487–2498. doi: 10.1002/hbm.20683, PMID: 19294641 PMC2827307

[ref13] Friederici A. D. (2017) Language in our brain: The origins of a uniquely human capacity; Camebridge, MA: London, UK.

[ref14] Gogos A. J. Young J. S. Morshed R. A. Hervey-Jumper S. L. Berger M. S. (2020). Awake glioma surgery: technical evolution and nuances. J. Neuro-Oncol. 147, 515–524. doi: 10.1007/s11060-020-03482-z, PMID: 32270374

[ref15] Grossman R. Nossek E. Sitt R. Hayat D. Shahar T. Barzilai O.. (2013). Outcome of elderly patients undergoing awake-craniotomy for tumor resection. Ann. Surg. Oncol. 20, 1722–1728. doi: 10.1245/s10434-012-2748-x 23212761

[ref16] Haddad A. F. Young J. S. Berger M. S. Tarapore P. E. (2021). Preoperative applications of navigated transcranial magnetic stimulation. Front. Neurol. 11:628903. doi: 10.3389/fneur.2020.628903, PMID: 33551983 PMC7862711

[ref17] Hartwigsen G. Volz L. J. (2021). Probing rapid network reorganization of motor and language functions via neuromodulation and neuroimaging. NeuroImage 224:117449. doi: 10.1016/j.neuroimage.2020.117449, PMID: 33059054

[ref18] Hervey-Jumper S. L. Berger M. S. (2016). Maximizing safe resection of low-and high-grade glioma. J. Neuro-Oncol. 130, 269–282. doi: 10.1007/s11060-016-2110-4 27174197

[ref19] Hervey-Jumper S. L. Li J. Lau D. Molinaro A. M. Perry D. W. Meng L.. (2015). Awake craniotomy to maximize glioma resection: methods and technical nuances over a 27-year period. J. Neurosurg. 123, 325–339. doi: 10.3171/2014.10.JNS141520 25909573

[ref20] Huber W. Poeck K. Springer L. (1983) Aachener Aphasietest (AAT), Göttingen: Hogrefe.

[ref21] Ille S. Engel L. Albers L. Schroeder A. Kelm A. Meyer B.. (2019). Functional reorganization of cortical language function in glioma patients-a preliminary study. Front. Oncol. 9:446. doi: 10.3389/fonc.2019.00446 31231608 PMC6558431

[ref22] Ille S. Krieg S. M. (2021). Functional mapping for glioma surgery, part 1: preoperative mapping tools. Neurosurg. Clin. N. Am. 32, 65–74. doi: 10.1016/j.nec.2020.08.004, PMID: 33223027

[ref23] Ille S. Schroeder A. Albers L. Kelm A. Droese D. Meyer B.. (2021). Non-invasive mapping for effective preoperative guidance to approach highly language-eloquent gliomas—a large scale comparative cohort study using a new classification for language eloquence. Cancers 13:207. doi: 10.3390/cancers13020207, PMID: 33430112 PMC7827798

[ref24] Ille S. Sollmann N. Butenschoen V. M. Meyer B. Ringel F. Krieg S. M. (2016). Resection of highly language-eloquent brain lesions based purely on rTMS language mapping without awake surgery. Acta Neurochir. 158, 2265–2275. doi: 10.1007/s00701-016-2968-0, PMID: 27688208

[ref25] Kertesz A. (2007) WAB-R: Western aphasia battery-revised, San Antonio, TX: The Psychological Corporation.

[ref26] Krieg S. M. Lioumis P. Mäkelä J. P. Wilenius J. Karhu J. Hannula H.. (2017). Protocol for motor and language mapping by navigated TMS in patients and healthy volunteers; workshop report. Acta Neurochir. 159, 1187–1195. doi: 10.1007/s00701-017-3187-z, PMID: 28456870

[ref27] Krieg S. M. Sollmann N. Hauck T. Ille S. Foerschler A. Meyer B.. (2013). Functional language shift to the right hemisphere in patients with language-eloquent brain tumors. PLoS One 8:e75403. doi: 10.1371/journal.pone.0075403, PMID: 24069410 PMC3775731

[ref28] Lemaitre A. L. Herbet G. Ng S. Moritz-Gasser S. Duffau H. (2022). Cognitive preservation following awake mapping-based neurosurgery for low-grade gliomas: a longitudinal, within-patient design study. Neuro-Oncology 24, 781–793. doi: 10.1093/neuonc/noab275, PMID: 34850187 PMC9071329

[ref29] Mandonnet E. Winkler P. A. Duffau H. (2010). Direct electrical stimulation as an input gate into brain functional networks: principles, advantages and limitations. Acta Neurochir. 152, 185–193. doi: 10.1007/s00701-009-0469-0 19639247

[ref30] Martin-Monzon I. Rivero Ballagas Y. Arias-Sanchez S. (2022). Language mapping: a systematic review of protocols that evaluate linguistic functions in awake surgery. Appl. Neuropsychol. Adult 29, 845–854. doi: 10.1080/23279095.2020.1776287 32543924

[ref31] Meier E. L. Sheppard S. M. Goldberg E. B. Head C. R. Ubellacker D. M. Walker A.. (2020). Naming errors and dysfunctional tissue metrics predict language recovery after acute left hemisphere stroke. Neuropsychologia 148:107651. doi: 10.1016/j.neuropsychologia.2020.107651, PMID: 33045231 PMC7546715

[ref32] Ojemann G. Ojemann J. Lettich E. Berger M. (1989). Cortical language localization in left, dominant hemisphere. An electrical stimulation mapping investigation in 117 patients. J. Neurosurg. 71, 316–326. doi: 10.3171/jns.1989.71.3.0316 2769383

[ref33] Oldfield R. C. (1971). The assessment and analysis of handedness: the Edinburgh inventory. Neuropsychol 9, 97–113. doi: 10.1016/0028-3932(71)90067-4 5146491

[ref34] Ostrom Q. T. Price M. Neff C. Cioffi G. Waite K. A. Kruchko C.. (2022). CBTRUS statistical report: primary brain and other central nervous system tumors diagnosed in the United States in 2015-2019. Neuro Oncol. 24, v1–v95. doi: 10.1093/neuonc/noac202 36196752 PMC9533228

[ref35] Penfield W. Roberts L. (1959) Speech and brain mechanisms, Princeton, NJ: Princeton University Press.

[ref36] Picht T. Kombos T. Gramm H. J. Brock M. Suess O. (2006). Multimodal protocol for awake craniotomy in language cortex tumour surgery. Acta Neurochir. 148, 127–138. doi: 10.1007/s00701-005-0706-0 16374563

[ref37] R Core Team (2022) R: A language and environment for statistical computing, Vienna, Austria: R Foundation for Statistical Computing.

[ref38] Rahimpour S. Haglund M. M. Friedman A. H. Duffau H. (2019). History of awake mapping and speech and language localization: from modules to networks. Neurosurg. Focus. 47:E4. doi: 10.3171/2019.7.FOCUS19347 31473677

[ref39] Robin X. Turck N. Hainard A. Tiberti N. Lisacek F. Sanchez J.-C.. (2011). pROC: an open-source package for R and S+ to analyze and compare ROC curves. BMC Bioinform. 12:77. doi: 10.1186/1471-2105-12-77, PMID: 21414208 PMC3068975

[ref40] Rstudio Team (2022) RStudio: integrated development environment for R, Boston, MA: RStudio, PBC.

[ref41] Ruis C. (2018). Monitoring cognition during awake brain surgery in adults: a systematic review. J. Clin. Exp. Neuropsychol. 40, 1081–1104. doi: 10.1080/13803395.2018.1469602, PMID: 30067443

[ref42] Sanchez-Pinto L. N. Venable L. R. Fahrenbach J. Churpek M. M. (2018). Comparison of variable selection methods for clinical predictive modeling. Int. J. Med. Inform. 116, 10–17. doi: 10.1016/j.ijmedinf.2018.05.006, PMID: 29887230 PMC6003624

[ref43] Sollmann N. Ille S. Boeckh-Behrens T. Ringel F. Meyer B. Krieg S. M. (2016). Mapping of cortical language function by functional magnetic resonance imaging and repetitive navigated transcranial magnetic stimulation in 40 healthy subjects. Acta Neurochir. 158, 1303–1316. doi: 10.1007/s00701-016-2819-z, PMID: 27138329

[ref44] Sollmann N. Kelm A. Ille S. Schroder A. Zimmer C. Ringel F.. (2018). Setup presentation and clinical outcome analysis of treating highly language-eloquent gliomas via preoperative navigated transcranial magnetic stimulation and tractography. Neurosurg. Focus. 44:E2. doi: 10.3171/2018.3.FOCUS1838, PMID: 29852769

[ref45] Surbeck W. Hildebrandt G. Duffau H. (2015). The evolution of brain surgery on awake patients. Acta Neurochir. 157, 77–84. doi: 10.1007/s00701-014-2249-8, PMID: 25352088

[ref46] Talacchi A. Santini B. Casagrande F. Alessandrini F. Zoccatelli G. Squintani G. M. (2013a). Awake surgery between art and science. Part I: clinical and operative settings. Funct. Neurol. 28, 205–221. doi: 10.11138/FNeur/2013.28.3.205, PMID: 24139657 PMC3812739

[ref47] Talacchi A. Santini B. Casartelli M. Monti A. Capasso R. Miceli G. (2013b). Awake surgery between art and science. Part II: language and cognitive mapping. Funct. Neurol. 28, 223–239. doi: 10.11138/FNeur/2013.28.3.223, PMID: 24139658 PMC3812745

[ref48] Thiel A. Habedank B. Winhuisen L. Herholz K. Kessler J. Haupt W. F.. (2005). Essential language function of the right hemisphere in brain tumor patients. Ann. Neurol. 57, 128–131. doi: 10.1002/ana.20342, PMID: 15622534

[ref49] Venables W. Ripley B. (2002) Modern applied statistics with S, 4th, New York: Springer

[ref50] Wang L. Chen D. Yang X. Olson J. J. Gopinath K. Fan T.. (2013). Group independent component analysis and functional MRI examination of changes in language areas associated with brain tumors at different locations. PLoS One 8:e59657. doi: 10.1371/journal.pone.0059657, PMID: 23555736 PMC3608667

[ref51] Wickham H. (2016) ggplot2: Elegant graphics for data analysis, New York: Springer-Verlag

[ref52] Youden W. J. (1950). Index for rating diagnostic tests. Cancer 3, 32–35. doi: 10.1002/1097-0142(1950)3:1<32::AID-CNCR2820030106>3.0.CO;2-3 15405679

[ref53] Yuen C. A. Barbaro M. Haggiagi A. (2022). Newly diagnosed glioblastoma in elderly patients. Curr. Oncol. Rep. 24, 325–334. doi: 10.1007/s11912-022-01201-7, PMID: 35122621 PMC8817659

[ref54] Zhang J. J. Y. Lee K. S. Voisin M. R. Hervey-Jumper S. L. Berger M. S. Zadeh G. (2020). Awake craniotomy for resection of supratentorial glioblastoma: a systematic review and meta-analysis. Neurooncol. Adv. 2:vdaa111. doi: 10.1093/noajnl/vdaa111, PMID: 33063012 PMC7542985

